# The Bonwill Electro-Magnetic Mallet

**Published:** 1881-07

**Authors:** 


### EDITORIAL, ETC.

---

*The Bonwill Electro-Magnetic Mallet.*—After a year of presence in the office, some months of which time quietly hanging against the wall, with at best only occasional use, this little instrument begins to assume the air of a friendly acquaintance. Its tricks and cranky ways are better understood, and although not yet a familiar friend, we begin to feel that we may speak of it with some little knowledge, though far from complete, of its use. The patient study of its use, seems to be that of years, at least so as to become a master, if indeed the older members of the profession *can* be masters of these new things. The proverb of "teaching the old dog," will be remembered by many; and after the coming of spectacles most practitioners will agree with us, that it is harder to "pick up" new tricks.

I. A few points about the *battery* itself seems worthy of comment. In it resides the mysterious soul of the instrument, and on its nice condition does success largely depend. If the zincs were called mercury-zinc amalgams, it would better explain the conditions required. Saturation of the zincs with mercury is a prerequisite of a steady current. A simple slight coating of mercury will not suffice. It must be poured on and poured on until no more is taken in, and then after a little, more added, and more mercury from time to time. One can't make the mistake of using too much, and too little is to fail of a steady current. Saturate the zincs until the finger-nail can readily scrape away the amalgamated surface of the metal, and in a few days it will be found that a good deal more can be added. All the bearings and connections must be clean. If a small file or strip of emery paper is passed through the hole in the binding post of the zinc to brighten the brass it will help greatly, and the copper wires themselves should be scraped bright when they pass into these holes. The screws themselves need looking after to see that they are tight. In a word intimate contact is essential to a steady current, and oxydation of surface means partial insulation.

II. Do not be afraid of the trouble of changing battery fluids often. As a good current is essential, and as it costs less to pay for a little sulphuric acid than to pay in vexation for a failure on a filling, so change fluids *before*, not after, the current shews signs of weakening. Better not trust a boy to do this; his sense of responsibility is not sufficiently great to keep "battery fluid" and dilute acid (porous cell solution and outside cell fluid) rigorously apart; a few drops of the inside fluid may not *seem* a matter of importance, but it will always do harm.

III. When the "fit" comes on your mallet don't go to turning the *adjusting screws*. If these are once set right they had best be left so; and you may safely conclude that the trouble is not in the mallet or its adjustments, but further off—in the *battery*. If you keep your solutions already mixed, a minute or two will serve to re-charge two cells, and this is most likely what is needed. Unless you can get the mallet to run smoothly and nicely you had better hang it up until you can get time to go over the case carefully, as it is impossible to make a good filling with any hitch in its working. Chemically pure acid seems to do rather better than the commercial—the battery will run longer without change; still the pure acid is not essential.

IV. Do not take it for granted that because it is an instrument moved by eclectricity and operated at immense speed, that therefore it will do your thinking for you. It is the intelligent hand holding the instrument that is the main factor of success. A careless operator may readily be made more so by relying on a machine. Its very rapidity renders it all the more essential that its action should be closely watched. Those who have witnessed Marshall H. Webb, who by common consent is the apostle of this instrument, will remember that he scrutinises the work with the utmost vigilance in all its stages. This is all the more important on account of the tendency of the instrument of light, quick blows, to "bridge over."

V. You cannot "shoot around the corner" with the electro-magnetic mallet. Operate on straight lines only. "Make your cavity accessible," is a maxim ever to be kept in mind. If you have been accustomed to soft foil and hand-pressure you are apt to forget this, but it is of essential importance.

It is hoped that these notes of our experience with this instrument may prove of some value to those who contemplate using it. It is certain that the purchase of an electro-magnetic mallet will not make a successful operator out of a careless or bungling manipulator. It requires on the other hand exceeding watchfulness and delicacy, but for those who do mallet work, it certainly saves a great deal of labor. The patient grumbles sometimes and sometimes does not.

